# On Snake-Poison: Its Action and Its Antidote

**Published:** 1894-03

**Authors:** 


*On Snake-Poison: its Action and its Antidote.* By A. MUELLER, M.D. Pp. 85. Sydney: L. BRUCK. 1893.

A portrait of the illustrious author, who describes himself as "an obscure Australian country practitioner" who "succeeded in a discovery for which all his predecessors in this field of research had laboured in vain," adorns this book. As we have made one quotation, we will indulge in a few more.

"By our united efforts"—for "our" read Dr. Mueller and "his Australian confrères," especially the former—"we have reared in a dark and hitherto barren field of research a column of solid knowledge, and on this column Australia now occupies the highest and will ever occupy the most prominent place." We sincerely hope that she will, and that the new Simon Stylites will be comfortable; meanwhile we confess a curiosity as to the nature of solid knowledge and of the utility of a column in a barren field.

Speaking of his theory that snake-poison paralyses the motor centres, the writer modestly remarks: "This and this only is the condition of the centres, the whole secret of snake-poison that has puzzled the human mind for ages, and yet appears so simple when discovered at last." "Its successful application (*i.e.* of strychnine) as the long and vainly sought antidote to snake-poison are glorious triumphs of scientific deduction."

We expect from an author with an elaborate theory of the action of snake-poison on the nervous system more knowledge of the elements of Physiology than is shown here. He is further not conversant with the latest observations on the chemical constitution of snake-venom, and we are entirely at issue with him when he concludes that chemical and experimental investigation on the subject is valueless. Future research in this direction will probably, as in the case of diphtheria, throw the greatest light on what we must still regard, in spite of the author's all-sufficient theories, as at present a badly-understood subject.

The author should also have shown, which he has made no attempt to do, that the bites of the Australian snakes are generally, or even often, dangerous to life—from other sources we gather that death from snake-bite is rare in Australia, especially in the southern parts of the continent. It does not follow that because patients recover from snake-bites after injection of strychnine, that they would not have recovered without it: at any rate, more evidence is required than he brings forward. We do learn one thing from his observations; and that is, that heroic doses of strychnine seem to be tolerated by persons suffering from snake-bites.

